# Supplementary material for: Antibacterial Effect of Sesame Protein-Derived Peptides against Escherichia coli and Staphylococcus aureus: In Silico and In Vitro Analysis
Source: Nutrients. 2024 Jan 4;16(1):175. doi: 10.3390/nu16010175 (PMC10780390; doi:10.3390/nu16010175)
Supplement: Supplementary file 1 [file nutrients-16-00175-s001.zip › nutrients-2733192-supplementary.pdf]

Supplementary materials

Table S1. UniProtKB id, number of amino acids and molecular mass of sesame protein.

Proteins	UniProtKB id and entry name	Amino acid residues	Molecular mass (Da)
11S globulin seed storage protein 2	Q9XHP0 (11S2_SESIN)	459	51840
2S seed storage protein 1	Q9XHP1(2SS1_SESIN)	148	17460

Table S2. The total number of ABPs peptides released from sesame protein hydrolysates by different proteases.

Sesame protein	Pepsin + trypsin + chymotrypsin A	Total peptides/composed of 2-15 AA/ABPs
11S globulin seed storage protein 2	PTC	169/106/25
2S seed storage protein 1	PTC	60/36/11

Table S3. Physicochemical properties of ABPs using in silico predictions.

S.no.	11S globulin seed storage protein 2/peptides	Net charge	Hydrophobic ratio	pI	MW (Da)	Water solubility
1	VAF	0	0.47	5.88	335.43	Poor water solubility
2	AL	0	0.39	5.88	202.27	Poor water solubility
3	QCAGIVAM	0	0.19	5.85	792.09	Poor water solubility
4	STIR	1	-0.37	10.11	475.59	Good water solubility
5	GL	0	0.35	5.88	188.25	Poor water solubility
6	PSPR	1	-0.54	10.11	455.55	Good water solubility
7	GQGL	0	0.04	5.88	373.47	Poor water solubility
8	ISIM	0	0.36	5.88	462.66	Poor water solubility
9	SQR	1	-0.9	10.11	389.44	Good water solubility
10	TM	0	0.04	5.88	250.33	Poor water solubility
11	GSVR	1	-0.33	10.11	417.51	Good water solubility
12	DL	-1	-0.09	3.8	246.28	Good water solubility
13	AF	0	0.43	5.88	236.28	Poor water solubility
14	AGGVPR	1	-0.12	10.11	555.71	Good water solubility
15	IF	0	0.67	5.88	278.37	Poor water solubility

16	IVM	0	0.51	5.88	361.54	Poor water solubility
17	PIL	0	0.4	5.88	341.49	Poor water solubility
18	SM	0	0	5.88	236.3	Good water solubility
19	VTR	1	-0.47	10.11	374.47	Good water solubility
20	GQAL	0	0.06	5.88	387.49	Poor water solubility
21	GF	0	0.39	5.88	222.26	Poor water solubility
22	EW	-1	-0.12	4	333.36	Good water solubility
23	AM	0	0.26	5.88	220.3	Poor water solubility
24	PL	0	0.23	5.88	228.31	Poor water solubility
25	GSQSF	0	-0.09	5.88	524.59	Poor water solubility

S.no.	2S seed storage protein 1/peptides	Net charge	Hydrophobic ratio	pI	MW (Da)	Water solubility
1	AAAL	0	0.32	5.88	344.45	Poor water solubility
2	CM	0	0.15	5.85	252.36	Poor water solubility
3	QW	0	-0.16	5.88	332.38	Poor water solubility
4	SM	0	0	5.88	236.3	Good water solubility
5	EH	-1	-0.51	5.25	284.29	Good water solubility
6	EL	-1	-0.04	4	260.31	Good water solubility
7	QM	0	-0.21	5.88	277.36	Poor water solubility
8	GM	0	0.21	5.88	206.28	Poor water solubility
9	QQM	0	-0.37	5.88	405.51	Poor water solubility
10	CGM	0	0.15	5.85	309.43	Poor water solubility
11	PIF	0	0.42	5.88	375.5	Poor water solubility

Table S4. Predicted toxicity, sensitization and Tanimoto similarity index using ABPs proteins from UniProtKB and NCBI databases.

11s globulin/ABPs	ToxicityPrediction	allergenicity	Tanimoto Similarity
VAF	Non-Toxin	PROBABLE NON-ALLERGEN	UniProtKB accession number P80820

AL	Non-Toxin	PROBABLE NON-ALLERGEN	UniProtKB accession number Q8IZT6
QCAGIVAM	Non-Toxin	PROBABLE NON-ALLERGEN	UniProtKB accession number P80813
STIR	Non-Toxin	PROBABLE NON-ALLERGEN	UniProtKB accession number Q8IZT6
GL	Non-Toxin	PROBABLE ALLERGEN	NCBI gi number 51316200
PSPR	Non-Toxin	PROBABLE NON-ALLERGEN	UniProtKB accession number P80820
GQGL	Non-Toxin	PROBABLE NON-ALLERGEN	UniProtKB accession number P80820
ISIM	Non-Toxin	PROBABLE ALLERGEN	NCBI gi number 51316200
SQR	Non-Toxin	PROBABLE NON-ALLERGEN	UniProtKB accession number P80820
TM	Non-Toxin	PROBABLE NON-ALLERGEN	UniProtKB accession number Q8IZT6
GSVR	Non-Toxin	PROBABLE NON-ALLERGEN	UniProtKB accession number P80820
DL	Non-Toxin	PROBABLE NON-ALLERGEN	UniProtKB accession number Q8IZT6
AF	Non-Toxin	PROBABLE ALLERGEN	NCBI gi number 51316200
AGGVPR	Non-Toxin	PROBABLE NON-ALLERGEN	UniProtKB accession number Q8IZT6
IF	Non-Toxin	PROBABLE NON-ALLERGEN	UniProtKB accession number Q8IZT6
IVM	Non-Toxin	PROBABLE ALLERGEN	NCBI gi number 51316200
PIL	Non-Toxin	PROBABLE ALLERGEN	NCBI gi number 51316200
SM	Non-Toxin	PROBABLE NON-ALLERGEN	UniProtKB accession number Q8IZT6
VTR	Non-Toxin	PROBABLE NON-ALLERGEN	UniProtKB accession number P80820
GQAL	Non-Toxin	PROBABLE ALLERGEN	NCBI gi number 51316200
GF	Non-Toxin	PROBABLE NON-ALLERGEN	UniProtKB accession number Q8IZT6
EW	Non-Toxin	PROBABLE NON-ALLERGEN	UniProtKB accession number Q8IZT6
AM	Non-Toxin	PROBABLE NON-ALLERGEN	UniProtKB accession number Q8IZT6
PL	Non-Toxin	PROBABLE NON-ALLERGEN	UniProtKB accession number Q8IZT6
GSQSF	Non-Toxin	PROBABLE ALLERGEN	NCBI gi number 51316200

**2s globulin/ABPs**	**Toxicity Prediction**	**Allergenicity prediction**	**Tanimoto Similarity**
AAAL	Non-Toxin	NON-ALLERGEN	UniProtKB P80820
CM	Non-Toxin	NON-ALLERGEN	UniProtKB Q8IZT6

QW	Non-Toxin	NON-ALLERGEN	UniProtKB Q8IZT6
SM	Non-Toxin	NON-ALLERGEN	UniProtKB Q8IZT6
EH	Non-Toxin	NON-ALLERGEN	UniProtKB Q8IZT6
EL	Non-Toxin	NON-ALLERGEN	UniProtKB Q8IZT6
QM	Non-Toxin	NON-ALLERGEN	UniProtKB Q8IZT6
GM	Non-Toxin	NON-ALLERGEN	UniProtKB Q8IZT6
QQM	Non-Toxin	PROBABLE ALLERGEN	NCBI gi number 51316200
CGM	Non-Toxin	NON-ALLERGEN	UniProtKB P80820
PIF	Non-Toxin	NON-ALLERGEN	UniProtKB P80821
ABPs	Toxicity Prediction	Allergenicity prediction	Tanimoto Similarity
